# Effects of Fermented Feed Supplementation on Production Performance and Egg Quality Parameters in Laying Hens: A Meta-Analysis

**DOI:** 10.3390/ani16060906

**Published:** 2026-03-13

**Authors:** Özge Sızmaz, Mohamed Tharwat, Muhammad Shazaib Ramay, Atakan Bundur, Muhammad Waqas, Hafiz Muhammad Nouman, Beenish Imtiaz, Ibrar Ahmed, Umair Ahsan, Fahad A. Alshanbari

**Affiliations:** 1 Department of Animal Nutrition and Nutritional Diseases, Faculty of Veterinary Medicine, Ankara University, Ankara 06110, Türkiye; ozgeabacioglu@gmail.com (Ö.S.); shazaibramay_7sky@yahoo.com (M.S.R.); atakanbundur@gmail.com (A.B.); 2 Department of Clinical Sciences, College of Veterinary Medicine, Qassim University, P.O. Box 6622, Buraidah 51452, Saudi Arabia; atieh@qu.edu.sa; 3 Department of Poultry Production, Faculty of Animal Production and Technology, University of Veterinary and Animal Sciences, Lahore 54000, Pakistan; 4 Department of Animal Nutrition and Nutritional Diseases, Faculty of Veterinary Medicine, Selçuk University, Konya 42130, Türkiye; drnomanqureshi@gmail.com (H.M.N.); vetrao6@gmail.com (I.A.); 5 Department of Animal Nutrition and Nutritional Disease, Faculty of Veterinary Medicine, Atatürk University, Erzurum 25240, Türkiye; beenish.imtiaz007@gmail.com; 6 Department of Plant and Animal Production, Burdur Vocational School Food, Agriculture and Livestock, Burdur Mehmet Akif Ersoy University, İstiklal Campus, Burdur 15030, Türkiye; umair.asawar46@gmail.com; 7 Department of Medical Biosciences, College of Veterinary Medicine, Qassim University, P.O. Box 6622, Buraidah 51452, Saudi Arabia

**Keywords:** egg production, egg quality, feed efficiency, fermented feed, laying hens, meta-analysis, systematic review

## Abstract

Fermentation-based feed processing has been proposed as a nutritional approach to improve nutrient availability and metabolic efficiency in laying hens. However, information on its impact on production performance and egg quality remains limited. This meta-analysis statistically assessed the impact of fermented feed supplementation on the production performance and egg quality parameters of laying hens while investigating potential sources of heterogeneity across studies. A comprehensive literature search was performed in PubMed, Scopus, and Web of Science using the PRISMA 2020 guidelines. The inclusion criteria were randomized controlled trials and controlled intervention studies with non-fermented control diets. Two primary outcomes were examined: (1) production performance and (2) egg quality. Multilevel random-effects meta-analyses employing restricted maximum likelihood were conducted to address multiple effect sizes. Heterogeneity was evaluated using Cochran’s Q statistic and I^2^ estimates. Meta-regression analyses were performed considering dietary inclusion level, trial duration, and total hens, with subgroup analyses based on fermented feed type, assessment of publication bias using Egger’s test and trim-and-fill methods, and leave-one-study-out sensitivity analysis. Twenty-four studies were included in the quantitative synthesis. Supplementation with fermented feed markedly enhanced the egg-laying rate (MD = 2.11 percentage points; 95% CI: 0.92–3.30; *p* = 0.0005), Haugh unit (MD = 1.99; 95% CI: 0.61–3.38; *p* = 0.0048), and eggshell thickness (MD = 0.0081 mm; 95% CI: 0.0037–0.0124; *p* = 0.0003), whereas no significant overall effect was noted on the feed-to-egg ratio (MD = −0.0384 g feed/g egg; 95% CI: −0.0871–0.0103; *p* = 0.1218). Significant heterogeneity was observed across outcomes (I^2^ ≈ 73–93%). Subgroup analyses revealed notable feed-type-specific effects, whereas meta-regression indicated that dietary inclusion level is a significant moderator of the feed-to-egg ratio. Sensitivity analysis validated the robustness of the pooled estimates, and publication bias did not significantly influence the results. Supplementation with fermented feed was associated with improvements in egg-laying rate, albumen quality (Haugh unit), and eggshell thickness in laying hens. However, substantial heterogeneity across studies and variability in effect sizes indicate that responses may depend on feed type, inclusion level, and study conditions. These findings should therefore be interpreted with caution, and further well-designed and standardized trials are needed to confirm the consistency and magnitude of these effects.

## Simple Summary

Efficient egg production is crucial for a consistent supply of nutritious feed. However, laying hens often experience nutritional limitations, including reduced nutrient digestibility and bioavailability caused by anti-nutritional factors and suboptimal gut microbial balance that negatively impact productivity and egg quality. Fermented feeds, produced by treating feed components with beneficial microorganisms, have been proposed as a practical nutritional strategy to improve feed value and performance. This study systematically analyzed 24 controlled experiments to evaluate the effects of fermented feed supplementation on egg production and egg quality in laying hens. Overall, fermented feed supplementation was associated with a statistically significant increase in egg-laying rate (MD = 2.11 percentage points; 95% CI: 0.92 to 3.30) and Haugh unit (MD = 1.99; 95% CI: 0.61 to 3.38), indicating improved internal egg quality. A small but statistically significant increase in eggshell thickness (MD = 0.0081 mm; 95% CI: 0.0037 to 0.0124) was also observed, which may contribute to reduced egg breakage during handling and transport. In contrast, fermented feed supplementation was not associated with a statistically significant change in feed-to-egg ratio (MD = −0.0384 g feed/g egg; 95% CI: −0.0871 to 0.0103), and substantial heterogeneity was observed across studies, indicating variability in feed efficiency responses. Importantly, the magnitude of the benefits varied depending on the type of fermented feed and the level of its inclusion in the diet. These findings demonstrate that fermented feeds can contribute to improving laying performance and egg quality, but their effectiveness depends on careful selection and application.

## 1. Introduction

Modern commercial egg production systems require consistent laying performance, regular eggshell integrity, and internal egg quality [1]. However, these goals are often undermined by nutritional constraints and physiological pressures that destabilize the highly controlled processes of egg formation. Mineral imbalances, particularly inadequate dietary calcium, can impair shell deposition, bone metabolism, and overall productivity in laying hens [2,3,4]. Fermentation processes may enhance mineral bioavailability by degrading anti-nutritional factors such as phytates, thereby improving calcium absorption and utilization. This mechanism may partly explain the observed increase in eggshell thickness; however, variability in basal dietary calcium levels and fermentation conditions across studies may have contributed to the heterogeneity in shell-related outcomes [5,6,7]. The combination of inflammatory stress and nutrient deficiency has a significant negative impact on egg production performance as well as on eggshell and bone metabolism in laying hens, highlighting the sensitivity of egg formation to nutrient intake and systemic stress [8]. These findings suggest that meeting minimum nutrient requirements alone may not always ensure optimal or stable egg production and quality under modern intensive production systems. Therefore, nutritional strategies that enhance nutrient utilization and gut function may provide additional benefits beyond baseline dietary adequacy [9,10].

Different dietary approaches have been used to reduce production loss and maintain egg quality. Methods such as alternative protein sourcing and targeted nutrient supplementation have been evaluated and appear to be effective in addressing specific constraints. For example, the incorporation of insect-based protein increased feed efficiency without impairing laying performance and shell quality [11], whereas amino acid supplementation strategies directly improved albumen quality by regulating reproductive tract function and protein synthesis [12]. Likewise, mineral-enzyme complexes enhance mineral bioavailability, improving eggshell thickness and breaking strength [13]. While evidence of nutritional manipulation of discrete production or quality traits exists, these strategies generally target specific physiological pathways but do not overcome systemic limitations associated with conventional feedstuffs, such as bioavailability, anti-nutritional factors, and gastrointestinal efficiency. Thus, more focus has been placed on feed processing solutions that can improve the quality of metabolic response variables, potentially leading to greater overall improvements in nutrient utilization and gut function. Fermentation modifies the feed matrix via microbial enzymatic activity, resulting in compositional changes that may enhance nutrient utilization and generate functional metabolites [14]. Fermentation enzymatically degrades complex compounds, lowers the concentrations of detrimental antinutrients, and adds specific microbial metabolites that can improve gut health and metabolic efficiency [15]. Fermented soybean meal is an example in which fermentation has been shown to reduce antinutritional factors and to produce antioxidant, immunomodulatory, and probiotic peptides and enzymes; however, critical factors such as the selection of microbial strains and optimal processing conditions remain poorly characterized [16]. Additional evidence in poultry indicates that fermented feeds enhance digestibility and intestinal morphology, depending on the fermentation design and levels of inclusion [6,17]. In addition, there is mechanistic evidence that post-fermented feeds may fortify enterocyte morphology by promoting villus architecture and altering nutrigenomic profiles through increased nutrient transporter gene expression, with a biological rationale for the resulting gain in absorptive capacity and metabolic efficiency [18]. Altogether, these results lay the foundation for fermentation as a processing strategy that can address nutrient availability, gut integrity, and overall systemic robustness—key areas that support egg production and quality.

Although interest in the use and benefits of fermented feeds in poultry has increased, the reported effects on laying performance and egg quality vary across studies due to differences in fermented substrate type, inclusion level, feeding duration, and fermentation conditions. Improvement of egg production and feed efficiency has been observed for fermented byproduct feeds [19], while co-fermented tea residue diets showed significant enhancement of laying rate, Haugh unit, eggshell thickness, antioxidant capacity, and microbial profiles; however, recommendations regarding optimal inclusion levels remain constrained by the potential effects of bioactive compounds [20]. Despite variability in the extent of effects due to the inclusion rate and study design, egg production, albumen quality, nutrient digestibility, intestinal morphology, antioxidant status, and immune function parameters of laying hens were consistently elevated with fermented soybean meal [21]. Additionally, a further indication that some microbial-based feed interventions can markedly improve egg production indices and feed efficiency is a finding from a related meta-analysis that responses are dose-dependent [21]. Despite this growing yet heterogeneous evidence base, a quantitative synthesis is needed to determine the effects of fermented feed supplementation on laying performance and egg quality traits and to systematically examine variability across fermented substrates, inclusion levels, and fermentation strategies. We hypothesized that fermented feed supplementation would improve egg-laying rate, internal egg quality (Haugh unit), and eggshell thickness compared with conventional diets, and that the magnitude of these effects would be influenced by fermented substrate type, inclusion level, and study duration. Thus, the current meta-analysis was conducted to systematically review controlled feeding trials and to identify potential sources of between-study heterogeneity.

## 2. Materials and Methods

### 2.1. Search Strategy

A systematic literature search was conducted according to the Preferred Reporting Items for Systematic Reviews and Meta-Analyses (PRISMA) guidelines to identify studies investigating the effects of fermented feed supplementation on the production performance and egg quality parameters of laying hens [22]. The search was constructed to be as broad as possible, incorporating controlled vocabulary terms (MeSH) and free-text keywords for fermentation, layer hens, diet supplementation, production performance, and egg quality responses. Database-specific Boolean operators and field tags were used to conduct electronic searches in PubMed, Scopus, and Web of Science (WOS). The final queries for the search were as follows:

PubMed: (Fermentation[MeSH Terms] OR fermented feed[tiab] OR fermented diet[tiab] OR fermented feedstuff*[tiab] OR fermented feed supplementation[tiab]) AND (Chickens[MeSH Terms] OR laying hens[tiab] OR layer hens[tiab] OR egg laying hens[tiab]) AND (Animal Feed[MeSH Terms] OR dietary supplementation[MeSH Terms] OR feed supplementation[tiab] OR dietary intervention*[tiab]) AND (Egg Production[MeSH Terms] OR production performance[tiab] OR productive performance[tiab] OR growth performance[tiab] OR feed conversion ratio[tiab] OR egg mass[tiab]) AND (Egg Quality[MeSH Terms] OR egg quality parameter*[tiab] OR eggshell thickness[tiab] OR eggshell strength[tiab] OR albumen height[tiab] OR haugh unit[tiab] OR yolk color[tiab]).

Scopus: TITLE-ABS-KEY(fermented feed OR fermented diet OR fermented feedstuff* OR fermented feed supplementation OR fermentation) AND TITLE-ABS-KEY(laying hens OR layer hens OR egg laying hens) AND TITLE-ABS-KEY(feed supplementation OR dietary supplementation OR dietary intervention*) AND TITLE-ABS-KEY(production performance OR productive performance OR growth performance OR feed conversion ratio OR egg production OR egg mass) AND TITLE-ABS-KEY(egg quality OR egg quality parameter* OR eggshell thickness OR eggshell strength OR albumen height OR haugh unit OR yolk color).

WOS: TS = (fermented feed OR fermented diet OR fermented feedstuff* OR fermented feed supplementation OR fermentation) AND TS = (laying hens OR layer hens OR egg laying hens) AND TS = (feed supplementation OR dietary supplementation OR dietary intervention*) AND TS = (production performance OR productive performance OR growth performance OR feed conversion ratio OR egg production OR egg mass) AND TS = (egg quality OR egg quality parameter* OR eggshell thickness OR eggshell strength OR albumen height OR haugh unit OR yolk color).

The literature search was conducted on 18 December 2025, representing the most recent comprehensive search prior to data extraction and statistical analysis. No date restrictions were applied. All records from the three databases were imported into EndNote 21 (Clarivate Analytics, Philadelphia, PA, USA), and duplicates were identified and removed before screening [23].

### 2.2. Screening

To systematically and transparently select the studies included in the review, a multi-stage screening process was conducted in accordance with PRISMA 2020. Screening was performed sequentially at the title, abstract, and full-text levels. First, records were screened by title to identify potentially relevant studies. All records deemed possibly eligible were advanced to the abstract screening stage. In the second stage, abstracts were independently screened for relevance and eligibility, and studies meeting predefined criteria were considered for full-text review. The final stage involved assessing the full texts of the remaining studies to confirm their eligibility for quantitative synthesis. Three reviewers independently conducted screening at the title, abstract, and full-text stages. Disagreements were resolved through discussion and consensus, and no automation tools were used during the screening process. Screening decisions were consistently conducted and recorded at each phase of the selection process, resulting in the final set of studies included in the meta-analysis.

### 2.3. Inclusion Criteria

Studies were included in the meta-analysis if they met all eligibility criteria. Peer-reviewed studies published in English that examined laying chickens, layer hens, egg-laying chickens, or referred to poultry at the egg production stage were included. The main exposure was concentrated on fermented feed or fermentation-related feeding (fermented diet, fermented feedstuff, or fermented feed ingredient). Studies had to report at least one outcome relating to production or productive performance (egg production, egg mass, feed intake, feed conversion ratio) and egg quality parameters (eggshell traits, albumen quality, and yolk traits). Studies were eligible if they were primary research studies that used randomized controlled trial (RCT) designs or observational study designs (cohort, case–control, or cross-sectional). However, only studies providing sufficient quantitative data for valid effect size calculation were included in the meta-analysis. Following full-text screening, all studies meeting criteria for quantitative synthesis were randomized controlled trials. In addition, studies were required to report sufficient quantitative statistical data that could be extracted for at least one eligible outcome from the fermented-feed treatment and control groups, allowing valid effect-size calculation. Mean ± standard deviation or standard error, raw numerical data by group, reported effect estimates with sample sizes, or other formats that allow for a quantitative synthesis were acceptable data formats.

### 2.4. Exclusion Criteria

Studies that failed to meet the predefined eligibility criteria were excluded from the meta-analysis. Specifically, studies were excluded if the animal population was not a laying hen, layer hen, egg-laying chicken, or poultry in the egg production phase or if the intervention was not fermented feed or fermentation-based dietary supplementation. Studies in which no production or productive performance outcomes or egg quality parameters were reported were also excluded. Studies that used ineligible study designs, including reviews, meta-analyses, protocols, opinion articles, conference abstracts without sufficient data, and other non-primary research, were excluded. We also excluded studies that were not peer-reviewed, not in English, or that lacked sufficient quantitative data for extraction, rendering a valid effect size impossible to calculate. Studies were also excluded if any of the eligibility criteria were missing, vague, ambiguous, or could not be verified from the full text.

### 2.5. Data Extraction

Predefined, standardized extraction templates were used to extract data, ensuring consistency across the included studies. The study information was structured in predefined column formats with standardized variable naming, and all variables were entered in the exact forms reported in the original study, with NR used where information was not provided.

Initially, a fixed table format was used to extract the study characteristics, including study ID, study design, population description, total sample size (when explicitly stated), intervention description, comparator description, and outcome assessment. The information on populations was documented as species and production phase with age (weeks) when reported, but not assuming the production phase. Intervention information was recorded in the form of the type of fermented feed used, level of inclusion, and length of use, as reported either in part or entirely, where only the reported part was extracted. The only outcome variables that were limited were production performance and egg quality, which were explicitly evaluated. Second, a separate standardized table was used to extract fermentation conditions, such as study identifier, fermentation time, fermentation temperature, and starter culture(s)/microorganism(s) (Table 2). Fermentation time and temperature readings were reported as noted without unit conversion, starter cultures were reported by their scientific names, and semicolons separated multiple organisms. If either the fermentation temperature or culture was not mentioned, it was entered as NR. Third, the meta-analysis of quantitative data extraction was limited to four predefined eligible outcomes when reported: egg-laying rate, feed-to-egg ratio, Haugh unit, and eggshell thickness. Extraction for each eligible outcome proceeded in a predefined column order to sequentially capture the following: study ID, experimental unit, trial duration, hen strain, laying phase, housing system, sample structure, control diet, details of the intervention (fermented feed type, microorganism, inclusion level, and feeding form), outcome name and unit, measurement time point, and group-level quantitative data for both control and treatment arms (mean, SD/SE/SEM as reported, and sample size *n*). The measure of variability (SD, SE, or SEM) was recorded precisely as reported in each study and consistently across each outcome. When essential quantitative information required for effect size calculation (group means, measures of variability, or sample sizes) was missing, attempts were made to derive the necessary statistics from the reported data where possible (conversion of SE to SD). Studies lacking sufficient extractable data were excluded from quantitative synthesis. Data extraction was performed independently by three reviewers using predefined standardized templates, and any discrepancies were resolved through discussion and consensus.

### 2.6. Data Organization

The data obtained were tabulated by outcome type to enable a systematic quantitative synthesis. All eligible data were grouped into four outcome-specific datasets, each independent according to the predetermined outcomes: egg-laying rate, feed-to-egg ratio, Haugh unit, and eggshell thickness. There were 24 studies on egg-laying rate, 22 on feed-to-egg ratio, 20 on Haugh unit, and 18 on eggshell thickness. For each outcome, data were organized using a fixed and consistent layout with the following columns: Study ID, Effect ID, Experimental Unit, Trial Duration (days), Hen Strain, Laying Age (weeks), Housing System, Total Hens, Number of Groups, Replicates per Group, Control Diet, Fermented Feed Type, Microorganism(s), Inclusion Level (%), Outcome Unit, Control Mean (C_Mean), Control SE (C_SE), Control Sample Size (C_*n*), Treatment Mean (T_Mean), Treatment SE (T_SE), and Treatment Sample Size (T_*n*). The Outcome Unit column was applied only to egg-laying rate, feed-to-egg ratio, and eggshell thickness, whereas Haugh unit was treated as a unitless variable. An independent review of every outcome-specific data set was conducted to ensure internal consistency in definitions and units used for variables, as well as in statistical formatting. Each dataset was prepared and validated accordingly before performing the statistical analysis.

### 2.7. Risk of Bias Assessment

The risk of bias was assessed using the SYRCLE risk-of-bias tool, which provides a specific approach to addressing bias in animal intervention studies [24]. The assessment was conducted using a three-step approach. In the first step, the SYRCLE tool was selected as the sole standardized assessment tool for all included studies. Second, methodological information was extracted domain-wise for each study based on the previously defined domains of the SYRCLE tool: sequence generation (selection bias), baseline characteristics (selection bias), allocation concealment (selection bias), random housing (performance bias), blinding (performance bias), random outcome assessment (detection bias), blinding (detection bias), incomplete outcome data (attrition bias), selective outcome reporting (reporting bias), and other sources of bias. In the third step, SYRCLE criteria were used to assign risk-of-bias judgments for each domain, and an overall risk-of-bias judgment was determined for each study. The structured nature of this methodological process facilitated consistent, transparent, and methodologically sound assessment of bias across all included RCTs. The overall risk-of-bias judgment was determined using a predefined decision rule. Studies were classified as high risk if at least one domain was judged as high risk, as unclear risk if multiple domains were rated unclear without any high-risk domain, and as low risk only if all domains were judged as low risk. Risk-of-bias assessment was conducted independently by three reviewers. Any discrepancies in domain-level judgments were resolved through discussion and consensus to ensure consistency and methodological accuracy.

### 2.8. Certainty of Evidence Assessment

The certainty of evidence for each key outcome was evaluated using the GRADE framework in accordance with PRISMA 2020 recommendations. Because the included studies were randomized or controlled intervention trials, evidence was initially rated as high certainty and subsequently downgraded, when appropriate, across five domains: risk of bias (based on SYRCLE assessments), inconsistency (heterogeneity statistics), indirectness, imprecision (confidence interval width and inclusion of the null), and publication bias (funnel plots, Egger’s test, and trim-and-fill analysis). Certainty levels were classified as high, moderate, low, or very low and summarized in a Summary of Findings table (Table 3).

### 2.9. Statistical Analysis

Statistical analyses were performed in R software [25] (version 4.4.2) using the Metafor [26], readxl [27], dplyr [28], stringr [29], and writexl [30] packages. In all studies, variability measures expressed as standard errors (SE) and were transformed into standard deviations (SD) before analysis. All outcomes were reported on a standard scale (egg-laying rate (%), feed-to-egg ratio (g feed/g egg), eggshell thickness (mm), and Haugh unit (unitless), so effect sizes were calculated as mean differences (MDs). When SE was reported, SD was calculated using SE and the appropriate sample size. For each outcome, separate three-level multilevel random-effects meta-analyses were conducted to account for statistical dependence arising from multiple effect sizes nested within the same study [31]. Twenty-four studies contributed data to the analysis of egg-laying rate, 22 to the analysis of feed-to-egg ratio, 20 to the analysis of Haugh unit, and 18 to the analysis of eggshell thickness. To address statistical dependence arising from multiple effect sizes within a single study, multilevel models were fitted. We specified a three-level meta-analytic structure, with effect sizes nested within studies (random effects: effect ID within study ID). Restricted maximum likelihood (REML) was used to estimate model parameters. The Q test for residual heterogeneity was used to assess statistical heterogeneity [32], and the I^2^ statistic was used to approximately quantify heterogeneity [33], calculated as the proportion of total variance explained by heterogeneity variance components relative to total variance.

Meta-regression analyses with dietary inclusion level (%), trial duration, and total number of hens included as continuous moderators were performed to explore possible sources of heterogeneity. These moderators were selected a priori based on biological plausibility and methodological considerations, including potential dose–response effects (inclusion level), time-dependent adaptation (trial duration), and study-scale or precision-related variability (total number of hens). Subgroup analyses by fermented feed type were also conducted. Differences between subgroups were formally tested by including feed type as a categorical moderator in the multilevel model, and subgroup-specific pooled estimates were obtained by fitting separate multilevel models within each subgroup. To prevent unit-of-analysis errors arising from multiple effect sizes per study, publication bias was evaluated at the study level. Within-study effect sizes were first aggregated using inverse-variance weighting, followed by identification of publication bias using funnel plots, the Egger regression test, and trim-and-fill [34,35]. A sensitivity analysis using a leave-one-study-out approach was conducted to evaluate the robustness of the pooled estimates [36]. This procedure involved refitting the multilevel meta-analysis while sequentially excluding one study at a time to assess the overall influence of individual studies on the results. All primary meta-analyses were run at the effect ID level; publication bias assessment and leave-one-out analyses were performed at the study ID level. Statistical tests were two-sided, and results were statistically significant at *p* < 0.05.

## 3. Results

### 3.1. Study Selection

**Figure 1:**
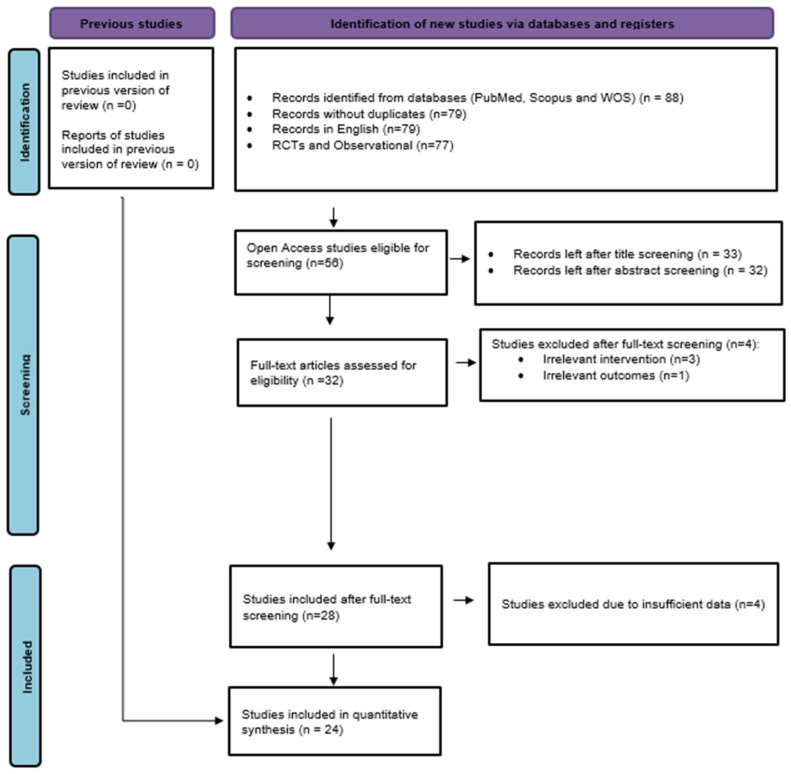
PRISMA flow diagram illustrating the identification, screening, eligibility assessment, and final inclusion of studies in the quantitative meta-analysis.

The study selection process was performed in accordance with the PRISMA 2020 guidelines to identify eligible studies transparently and systematically. A total of 88 records were generated from the electronic search through PubMed (n = 7), Web of Science (n = 81), and Scopus (n = 0). Nine duplicate records were removed (total n = 79 unique records remained). All the records were in English. After filtering records by study design, 77 records (randomized controlled trials or observational studies) remained, and two qualitative studies were excluded. Due to institutional access limitations, only studies with accessible full-text availability were screened at the eligibility stage. After applying this accessibility filter, 56 records proceeded to title screening. In the title screening, 23 records were excluded as not relevant, and 33 were selected for abstract screening. One study was excluded during abstract screening, leaving 32 studies available for full-text assessment. Eight studies were excluded during full-text screening. Among these, three studies were excluded because the intervention was not fermented feed [37,38,39], one study was excluded due to unrelated outcomes [40], and four studies were not extractable for quantitative synthesis [41,42,43,44]. After the application of all eligibility criteria, a total of 24 studies were eligible for inclusion and have therefore been included in the quantitative meta-analysis. The PRISMA flow diagram below (Figure 1) summarizes the study selection process.

### 3.2. Study Characteristics

The study characteristics provide an overview of all included studies and summarize key methodological and experimental details, including study design, characteristics of laying hen populations, sample size, fermented feed interventions, comparators, and production performance and egg quality outcomes assessed. Fermentation-related details, such as duration, temperature, and the species of microbes, are noted separately. This organization is presented in Table 1 and Table 2 to enable comparison between studies and to help explain heterogeneity within the summary effect estimates identified in the meta-analysis.

**Table 1:** Study Characteristics.

Study	Population	Sample Size	Intervention	Comparator	Outcomes
Chen, Zhou [20]	Lohmann laying hens, 34 weeks	1296	Tea residue-fermented feed, 1–5%, 6 weeks	Basal diet	Feed intake, egg-laying rate, average egg weight, feed-to-egg ratio, egg yolk color, albumen height, Haugh unit, eggshell thickness, egg shape index
Choi, Lee [45]	Hy-Line Brown layers, 70 weeks	180	Fermented brown seaweed by-product, 0.5%, fermented seaweed fusiforme by-product, 0.5%	Control (basal diet)	Feed intake, egg production rate, egg weight, egg mass, egg shell color, egg yolk color, egg shell strength, egg shell thickness, Haugh unit
Guo, Xu [46]	Laying hens (Hy-Line Brown), 80 weeks	360	Fermented feed, 20%, 10 weeks	Basal diet	Average daily feed intake; average egg weight; laying rate; broken egg rate; feed conversion ratio; eggshell thickness; eggshell weight; albumen height; Haugh unit; yolk weight; eggshell breaking strength; yolk colour
Jiang, Zhang [47]	Hy-Line Brown laying hens, late laying period, 390 days	360	Fermented Chinese herbal medicine, 2%, 8 weeks; enzymatically fermented Chinese herbal medicine, 2%, 8 weeks; crushed Chinese herbal medicine, 2%, 8 weeks	Basal diet	Laying rate; egg weight; feed intake; feed conversion ratio; Haugh unit; albumen height; yolk color; eggshell thickness; shell strength; egg shape index
Konkol, Popiela [48]	Lohmann Brown laying hens, 26 wk of life	108	Fermented rapeseed meal (FRSM), 3%, 90 d	Control group (no rapeseed meal); unfermented rapeseed meal (URSM), 3%	Egg production; egg weight; feed intake; feed conversion ratio; yolk color; Haugh Units; eggshell breaking strength; eggshell thickness
Kothari, Oh [49]	Laying hens (Hy-Line brown), 40 weeks of age	108	Fermented pine needle extract (FPNE), 2.5 mL/kg or 5 mL/kg, 6 weeks	Basal diet + 0 mL FPNE/kg diet	Egg production percentage; Egg mass; Feed intake; Egg weight; Feed conversion ratio; Eggshell color; Egg yolk color; Eggshell breaking strength; Haugh unit; Albumen height; Eggshell thickness
Lee, Niu [50]	Laying hens, 40 weeks old	180	Fermented *Artemisia annua* L. dried leaves, 0.5%, 6 weeks	Basal diet (CON)	Egg production rate; egg weight; egg mass; feed intake; feed conversion ratio; Haugh unit; eggshell color; egg yolk color; eggshell breaking strength; eggshell thickness
Lee, Moon [51]	Laying hens, 40 weeks old	120	Solid-state fermented Chinese chives, 0.5% or 1.5%, 5 weeks	Basal diet + carrier mixture	Egg production rate; average egg weight; egg mass; feed intake; feed conversion ratio; Haugh unit; eggshell breaking strength; eggshell thickness; egg yolk color
Li, Qin [52]	Laying hens, aged, 345 days	320	Fermented *Aronia melanocarpa* pomace, 0.25–1.0%, 8 weeks	Basal diet	Average laying rate; average egg weight; average daily feed intake; feed-to-egg ratio; egg shape index; eggshell thickness; eggshell strength; albumen height; Haugh unit; yolk color; egg weight; percentages of eggshell, yolk, and albumen
Liu, Jin [53]	Laying hens, 60 weeks	80	Spent ginger yeast cultures, 5–40 g/kg, 6 weeks	Basal diet	Laying rate; feed conversion ratio; average daily feed intake; average egg weight; albumen height; yolk color; Haugh unit; shell strength; shell thickness; egg shape index
Liu, He [54]	Laying hens, 34 weeks	1260	Fermented Chinese herbal compounds, 2% and 3%, 7 weeks	Diet without fermented Chinese herbal compounds	egg laying rate; average daily egg weight; average daily feed intake; feed–egg ratio; cracked shell egg rate; soft shell egg rate; yolk color; albumen height; Haugh unit score; eggshell strength; eggshell thickness; egg breaking rate; eggshell color; egg yolk cholesterol; egg yolk total triglyceride contents
Lu, Zeng [55]	Laying hens, 54 wk	1008	Fermented soybean meal, 2%, 4%, and 8%, 12 wk; Fermented miscellaneous meal (cottonseed meal:coconut meal = 1:1), 2%, 4%, and 8%, 12 wk	Corn-soybean-base diet	egg production rate; average egg weight; egg mass; average daily feed intake; feed conversion rate; egg shape index; eggshell strength; eggshell weight; eggshell thickness; egg yolk color; albumen height; Haugh unit
Lv, Guo [56]	Laying hens, 22 weeks	360	*Clostridium butyricum* fermented feed, 6%, 8 weeks; *Lactobacillus crispatus* fermented feed, 6%, 8 weeks; *Lactobacillus salivarius* fermented feed, 6%, 8 weeks	Basal diet (CON)	Laying rate; Average egg weight; Average daily feed intake (ADFI); Feed conversion ratio (FCR); Egg shape index; Eggshell thickness; Eggshell strength; Albumen height; Yolk color; Haugh unit
Niu, Wang [57]	HY-Line Brown laying hens, 23 weeks	288	*Lactobacillus plantarum*–fermented *Broussonetia papyrifera*, 1% or 5%, 56 d	Basal diet	Feed intake; feed conversion ratio; average egg weight; laying ratio; qualified egg ratio; egg yolk color; eggshell weight; eggshell thickness; eggshell strength; albumen height; albumen weight; albumen ratio; yolk weight; yolk ratio; Haugh unit
Qin, Li [58]	Yukou Jingfen No. 8 laying hens, late laying period, 345 d old	320	Fermented blueberry pomace, 0.25%, 0.5%, 1.0%, 56 d	Basal diet	Egg production; egg weight; average laying rate; average egg weight; average daily feed intake; feed-egg ratio; albumen height; egg yolk color; Haugh unit; eggshell thickness; egg shape index; egg white percent; egg yolk percent; eggshell percent; eggshell strength
Sun, Wu [59]	Laying hens (Lohmann Brown), 40 weeks	288	Red yeast rice, 0.5/1/5 g/kg, 8 weeks	Basal diet	Feed intake, egg weight, laying rate, feed conversion ratio, eggshell thickness, eggshell breaking strength, albumen height, Haugh units, yolk colour score, egg-yolk cholesterol, egg-yolk triglyceride
Tian, Li [60]	Xianju laying hens, 34 weeks old	120	Fermented plant product (FPP), 500 mg/kg, 8 weeks	Basal diet without FPP (CON)	Egg production; Egg mass; Feed intake; Feed conversion ratio (FCR); Egg weight; Egg shape index; Eggshell thickness; Eggshell breaking strength; Yolk color; Yolk index; Albumen height; Haugh unit
Trinh, Lee [61]	Lohmann Brown laying hens, 67 weeks old	60	Fermented mugwort and water spinach juice (FMW), 0.1% and 0.2%, 4 weeks	Control diet (basal diet)	Egg production rate; Average egg weight; Egg mass; Feed intake; Feed conversion ratio; Egg weight; Albumen height; Haugh unit; Yolk color; Eggshell strength; Eggshell thickness; Eggshell color; Yolk height
Wang, Zheng [62]	Hy-Line Brown laying hens, 18 weeks old	128	Fermented heat-treated rice bran, 2.5% or 5.0%, 8 weeks	Heat-treated rice bran (2.5% or 5.0%); basal diet	Average daily feed intake; egg production; average egg weight; feed conversion ratio
Xu, Li [63]	Laying hens, 24 weeks	180	Fermented cottonseed meal (FCSM), 45 days	Soybean meal (SBM) basal diet	Egg production; Egg laying rate; Average egg weight; Death rate during egg laying; Feed-to-egg ratio; Albumen height; Yolk color; Haugh unit; Eggshell thickness; Eggshell strength; Eggshell ratio
Xu, Yi [64]	Laying hens, 50 weeks old	288	Shuanghuanglian fermentation liquor (SFL) in drinking water (0.3%, 0.5%, 0.7%), 37 days	0% SFL in drinking water	Average egg weight; Laying rate; Average daily feed intake; Feed-to-egg ratio; Eggshell thickness; Albumen height; Yolk color; Egg shape index; Haugh unit
Yi, He [65]	laying hens, 50 weeks old	288	fermented *Artemisia annua* residue, 1%, 2%, 4%, 30 days	basal diet	average daily feed intake; egg weight; laying rate; feed-to-egg ratio; albumen height; Haugh unit; egg shape index; eggshell thickness; yolk color
Zheng, Oh [66]	Hy-Line Brown laying hens, 80 weeks	108	Fermented *Chlorella vulgaris* (CBT^®^), 0/1000/2000 mg/kg, 42 days	Basal diet	Feed intake; Egg production; Egg mass; Egg weight; Yolk color; Haugh unit; Eggshell thickness; Eggshell strength; Eggshell color
Zhu, Tao [67]	Laying hens, late laying period, 67 weeks	360	*Broussonetia papyrifera*–fermented feed, 1.5%, 3%, 4.5%, 56 days	Basal diet	Average daily feed intake; average egg weight; average daily egg production; laying rate; feed-to-egg ratio; egg shape index; eggshell colour; eggshell strength; albumen height; Haugh unit; yolk colour; eggshell thickness; relative weight of yolk; relative weight of eggshell

**Table 2:** Fermenting Conditions.

Study ID	Time	Temperature	Culture
Chen, Zhou [20]	96 h	36 °C	*B. subtilis*; *L. plantarum*
Choi, Lee [45]	48 h	37 °C	*Bacillus subtilis*
Choi, Lee [45]	96 h	37 °C	*Aspergillus oryzae*
Guo, Xu [46]	14 days	37 °C	*Bacillus subtilis*; *Saccharomyces cerevisiae*
Jiang, Zhang [47]	7 d	37 °C	*Lactobacillus plantarum*; *Saccharomyces cerevisiae*; *Aspergillus niger*
Konkol, Popiela [48]	24 h	37 °C	*Bacillus subtilis* 67 strain
Kothari, Oh [49]	6 months	room temperature	NR
Lee, Niu [50]	24 h	37 °C	*L. plantarum* SK3494
Lee, Moon [51]	24 h	30 °C	*L. plantarum* SK4719
Li, Qin [52]	NR	75 °C	*Bacillus subtilis* GDMCC 1.372; *Bacillus licheniensis* GDMCC 1.182; *Lactobacillus plantarum* GDMCC 1.648; *Rhodotorula benthica* GDMCC 2.215
Liu, Jin [53]	NR	20 °C	*S. cerevisiae*
Liu, He [54]	7 d	NR	*Bacillus subtilis*; *Bacillus coagulans*; *Enterococcus faecalis*; *Lactobacillus plantarum*; *Saccharomyces cerevisiae*
Lu, Zeng [55]	72 to 120 h	NR	*Bacillus*; *Saccharomyces*; *Lactobacillus*; *Clostridium butyricum*
Lv, Guo [56]	5 d	20 ± 5 °C	*C. butyricum*; *L. crispatus*; *L. salivarius*
Niu, Wang [57]	7 d	room temperature (∼30 °C)	*L. plantarum*
Qin, Li [58]	64 h	75 °C	*Bacillus subtilis*; *Bacillus licheniformis*; *Lactobacillus plantarum*; marine red yeast
Sun, Wu [59]	12 days	30 °C	*Monascus purpureus* GM-039
Tian, Li [60]	NR	NR	*Lactobacillus acidophilus*; *Bacillus subtilis*
Trinh, Lee [61]	24 h	30 °C	*Pediococcus pentosaceus*; *Lactiplantibacillus plantarum*; *Lactiplantibacillus pentosus*; *Pichia kudriavzevii*
Wang, Zheng [62]	36 h	35 °C	*Bacillus subtilis*; *Lactobacillus plantarum*
Xu, Li [63]	14 days	room temperature	*Bacillus culture*
Xu, Yi [64]	7 days	28 °C	*Bacillus amyloliquefaciens*-c4
Yi, He [65]	6 days	28 °C	*Bacillus amyloliquefaciens*-c4
Zheng, Oh [66]	72 h	NR	baker’s yeast; lactic acid bacterium
Zhu, Tao [67]	20 days	25–30 °C	*Enterococcus faecalis*

### 3.3. Risk of Bias Assessment

**Figure 2:**
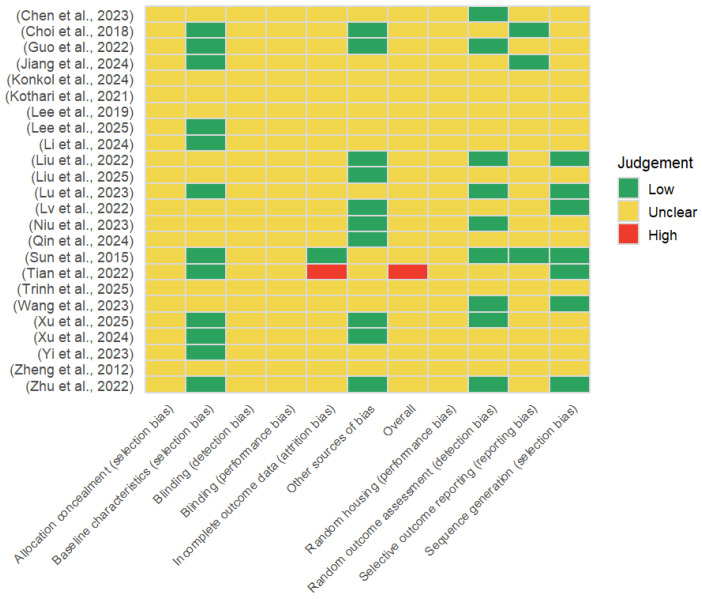
Summary of risk of bias assessments for the included randomized controlled animal studies using the SYRCLE risk-of-bias tool, showing the proportion of domain-level judgments classified as low risk, unclear risk, or high risk across all evaluated bias domains [20,45,46,47,48,49,50,51,52,53,54,55,56,57,58,59,60,61,62,63,64,65,66,67].

The SYRCLE risk-of-bias tool for randomized controlled trials was used to assess the risk of bias (RoB) of the studies included. All included studies were assessed according to SYRCLE’s prespecified domains, which were selection bias (sequence generation, baseline characteristics, allocation concealment), performance bias (random housing, blinding), detection bias (random outcome assessment, blinding), attrition bias (incomplete outcome data), reporting bias (selective outcome reporting), and other bias. Risks of bias judgments were made based on individual domains of bias risk, with each domain assessed as low, unclear, or high risk; for each study, a final overall risk-of-bias judgment was generated based on the composition of the domain-level assessments. Figure 2 presents domain-wise and overall RoB judgments per individual study and provides a high-level overview of the pervasive methodological quality across the included evidence base.

### 3.4. Certainty of Evidence

The certainty of evidence ranged from low to very low across outcomes (see Table 3). For egg-laying rate, certainty was rated as low due to serious risk-of-bias concerns and substantial heterogeneity. Feed-to-egg ratio was rated as very low because of serious heterogeneity, imprecision (confidence interval crossing the null), and overall risk-of-bias concerns. Haugh unit was also rated as very low due to very high heterogeneity, evidence of small-study effects, and methodological limitations of the included studies. Eggshell thickness was rated as very low owing to substantial inconsistency, instability in sensitivity analyses, and risk-of-bias concerns. Overall, confidence in the pooled estimates was primarily limited by methodological uncertainty and heterogeneity across studies.

**Table 3:** Summary of findings and certainty of evidence (GRADE).

Outcome	Studies	Pooled Effect (MD)	95% CI	Certainty (GRADE)
Egg-laying rate (%)	24	+2.11	0.92 to 3.30	Low
Feed-to-egg ratio (g/g)	22	−0.0384	−0.0871 to 0.0103	Very low
Haugh unit	k = 50 ES	+1.99	0.61 to 3.38	Very low
Eggshell thickness (mm)	k = 48 ES	+0.0081	0.0037 to 0.0124	Very low

### 3.5. Production Performance

#### 3.5.1. Egg Laying Rate

**Figure 3:**
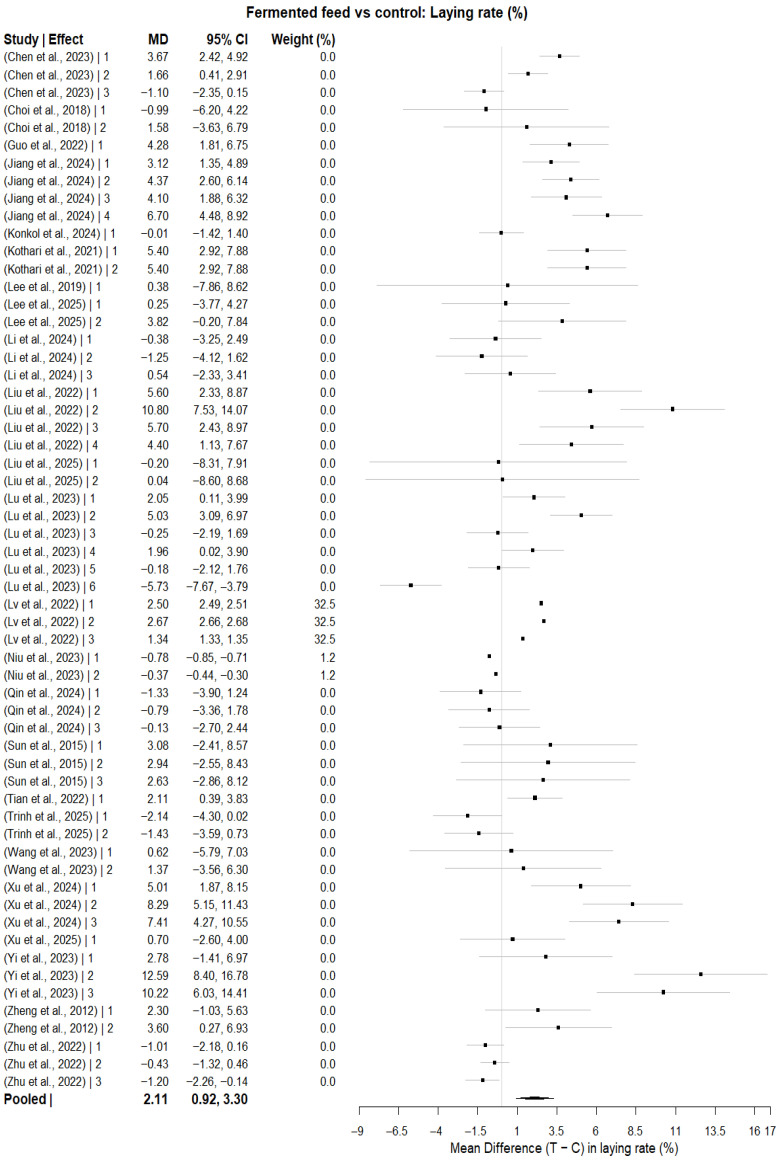
Forest plot showing pooled mean differences (MD) and 95% confidence intervals for the effect of fermented feed supplementation on egg-laying rate in laying hens under a multilevel random-effects model [20,45,46,47,48,49,50,51,52,53,54,55,56,57,58,59,60,61,62,63,64,65,66,67]. Squares indicate the effect size estimate for individual studies (with the area of squares proportional to study weight), and horizontal lines indicate corresponding 95% confidence intervals. The diamond represents the pooled effect estimate.

A multilevel random-effect meta-analysis based on 59 effect sizes obtained from 24 studies estimated a statistically significant pooled MD for egg-laying rate between fermented-feed and control groups (MD = 2.11 percentage points; 95% CI: 0.92 to 3.30; *p* = 0.0005), as shown in Figure 3. Heterogeneity was evident (Q = 32,745.70, df = 58, *p* < 0.0001). The total between-effect variance (τ^2^) was 9.12, of which 65.99% of the total variance was attributable to between-study heterogeneity and 34.01% was the within-study (effect-level) variance. The estimated heterogeneity was I^2^ = 73.83% (based on mean sampling variance) and 84.17% (based on median sampling variance). The high I^2^ values indicate substantial between-study variability, suggesting that pooled estimates should be interpreted cautiously. Meta-regression analyses indicated that dietary inclusion level (%), trial duration, and total number of hens were not statistically significant moderators of the effect size (all *p* > 0.05).

**Figure 4:**
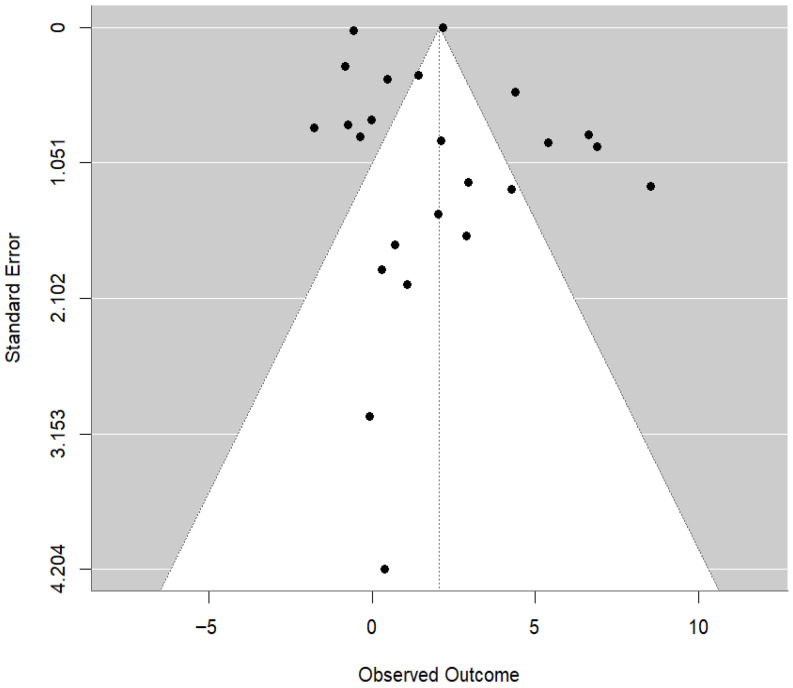
Funnel plot of study-level effect sizes for egg-laying rate, showing the relationship between observed outcomes and standard error to assess potential publication bias. Each dot represents the effect size of an individual study. The pooled effect estimate is represented by the vertical dotted line, and the shaded triangular region represents the expected 95% confidence limits around the pooled estimate.

Subgroup analysis using fermented feed type as a categorical moderator (k = 31 effect sizes) identified a statistically significant subgroup difference (QM = 37.90, df = 9, *p* < 0.0001), although residual heterogeneity remained significant (QE = 99.70, df = 21, *p* < 0.0001). Compared with the reference feed type, statistically significant pooled mean differences were observed for fermented *Artemisia annua* residues (AAR) (MD = 9.41; 95% CI: 4.40 to 14.41; *p* = 0.0002), SGYCs (MD = 7.50; 95% CI: 2.99 to 12.02; *p* = 0.0011), and Shuanghuanglian fermentation liquor (SFL) (MD = 7.78; 95% CI: 3.04 to 12.52; *p* = 0.0013), whereas other fermented feed types did not show statistically significant differences (*p* > 0.05). In the leave-one-study-out sensitivity analysis, the combined MD estimates ranged from 1.82 to 2.30 across all study exclusions, and the associated 95% CI remained above zero for each study, suggesting no instability in the combined effect. There was no evidence of funnel plot asymmetry (Figure 4), as also suggested by the Egger’s regression test at the study-level summary effect for publication bias (z = 0.2440, *p* = 0.8072).

#### 3.5.2. Feed-to-Egg Ratio

**Figure 5:**
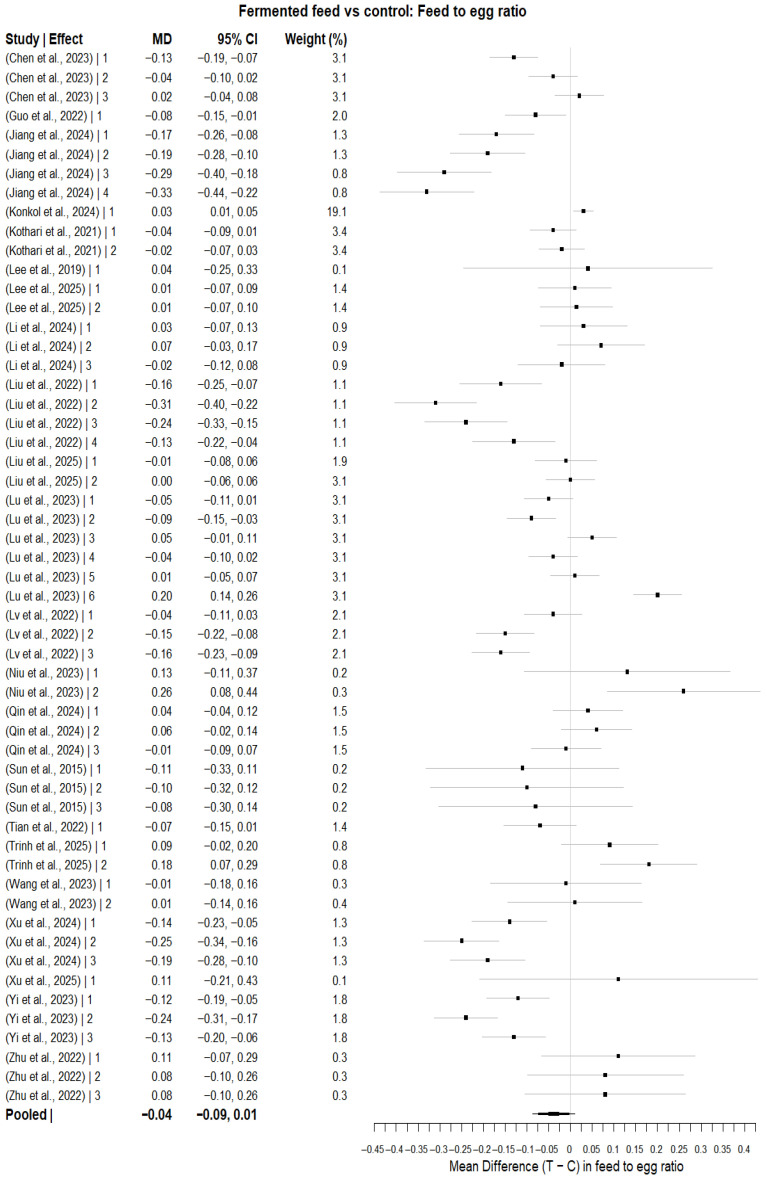
Forest plot showing pooled mean differences (MD) and 95% confidence intervals for the effect of fermented feed supplementation on feed-to-egg ratio in laying hens under a multilevel random-effects model. Squares indicate the effect size estimate for individual studies (with the area of squares proportional to study weight), and horizontal lines indicate corresponding 95% confidence intervals. The diamond represents the pooled effect estimate [20,46,47,48,49,50,51,52,53,54,55,56,57,58,59,60,61,62,63,64,65,67].

**Figure 6:**
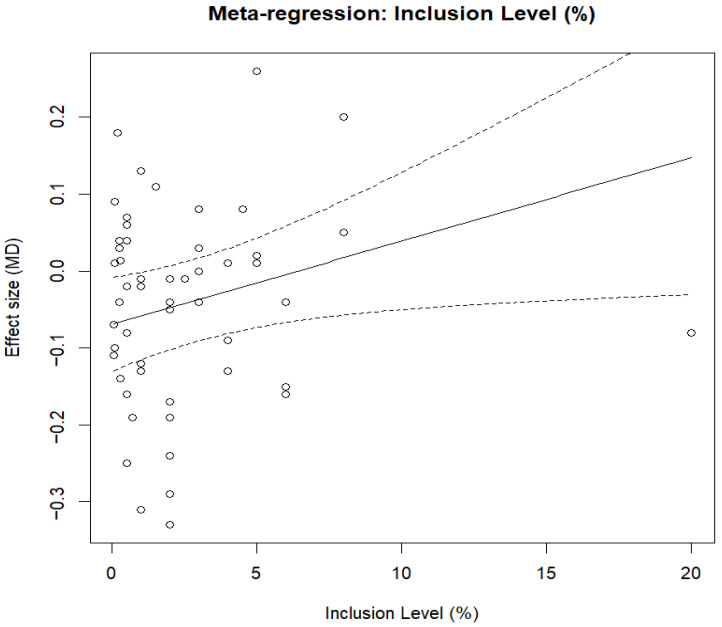
Meta-regression plot illustrating the relationship between fermented feed inclusion level (%) and effect size (mean difference) for feed-to-egg ratio in laying hens. The fitted meta-regression line is shown with the solid line, while the 95% confidence bands around the regression estimate based on the multilevel random-effects model are indicated by dashed lines.

For the feed-to-egg ratio (g feed/g egg), the multilevel random-effects meta-analysis was based on 54 effect sizes from 22 studies and revealed a pooled mean difference of MD = −0.0384 (CI 95%: −0.0871 to 0.0103; *p* = 0.1218), as presented in Figure 5. There were statistically significant heterogeneity effects (Q = 437.37, df = 53, *p* < 0.0001). The estimated τ^2^ values were 0.009829 (between-study) and 0.003605 (within-study), yielding a total τ^2^ of 0.013434, with between-study variance accounting for 73.17% and within-study variance accounting for the remaining 26.83%. The respective I^2^ values were 77.12% (based on mean sampling variance) and 87.48% (based on median sampling variance). The high I^2^ values indicate substantial between-study variability, suggesting that pooled estimates should be interpreted cautiously. The meta-regression (Figure 6) evaluating inclusion level (%) was statistically significant (QM = 4.6260, df = 1, *p* = 0.0315), with a slope estimate of MD = 0.0109 per 1% increase in inclusion level (95% CI: 0.0010 to 0.0207; *p* = 0.0315). Centered inclusion level yielded the same slope estimate (Incl_c = 0.0109, CI: 0.0010–0.0207; *p* = 0·0315). In the assessment of a quadratic dose–response relationship, the moderator set was statistically significant (QM = 9.0035, df = 2, *p* = 0.0111) with Incl = 0.0299 (95% CI: 0.0085 to 0.0513; *p* = 0.0061) and Incl^2^ = −0.0013 (*p* = 0569); comparison of quadratic vs. linear model LRT: LRT = −1.1441, *p* = 2848.

For the subgroup analysis (feed-to-egg ratio), fermented feed type was associated with significant differences in effect sizes (QM = 32.9978, df = 9, *p* = 0.0001); however, residual heterogeneity remained significant (QE = 88.8360, df = 21, *p* < 0.0001). Compared with the reference feed type, significant reduction in feed-to-egg ratio was observed for fermented *Artemisia annua* residues (AAR) (estimate = −0.2536, 95% CI: −0.4293 to −0.0780, *p* = 0.0047), SGYCs (estimate = −0.3003, 95% CI: −0.4735 to −0.1271, *p* = 0.0007), and Shuanghuanglian fermentation liquor (SFL) (estimate = −0.2836, 95% CI: −0.4613 to −0.1059, *p* = 0018), whereas no significant decrease was observed for the other fermented feeds. The overall moderator test was significant in the adjusted multilevel model for level of inclusion and fermented feed type (QM = 121.3502, df = 25, *p* < 0.0001). Inclusion level continued to be a strong predictor (estimate = 0.0274, *p* < 0.0001), and several fermented feeds (SGYCs, SFL, and various types of fermented CHM) showed significant subgroup effects. In contrast, others did not, indicating heterogeneity in feed-specific responses beyond dosage alone.

**Figure 7:**
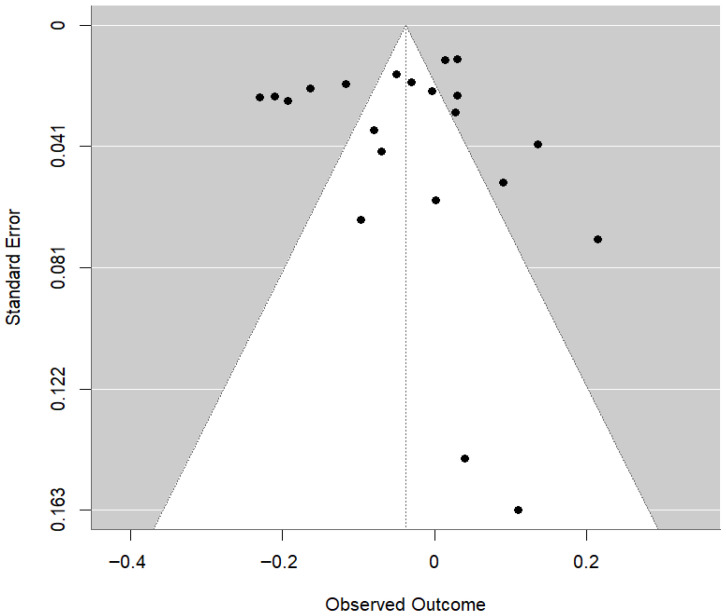
Funnel plot of study-level aggregated effect sizes for feed-to-egg ratio, depicting the relationship between observed outcomes and standard error to assess potential publication bias. Each dot represents the effect size of an individual study. The pooled effect estimate is represented by the vertical dotted line, and the shaded triangular region represents the expected 95% confidence limits around the pooled estimate.

Sensitivity analysis using the leave-one-study-out strategy showed that pooled estimates ranged from −0.0486 (omitting [57]) to −0.0282 (omitting [47]), with confidence intervals fully encompassing the central pooled estimate. However, the exclusion of Niu, Wang [57] generated a confidence interval that just barely avoided zero (95% CI = −0.0953 to −0.0019), and the direction and strength of the effect were preserved, thus showing the robustness of the results. No significant publication bias was suggested by the funnel plot about the pooled results of studies (Figure 7) or Egger’s regression test (z = 1.6860, *p* = 0.0918). The estimated Egger intercept was −0.0893 (95% CI, −0.1649 to −0.0137), with no firm evidence of small-study effects.

### 3.6. Egg Quality

#### 3.6.1. Haugh Unit

**Figure 8:**
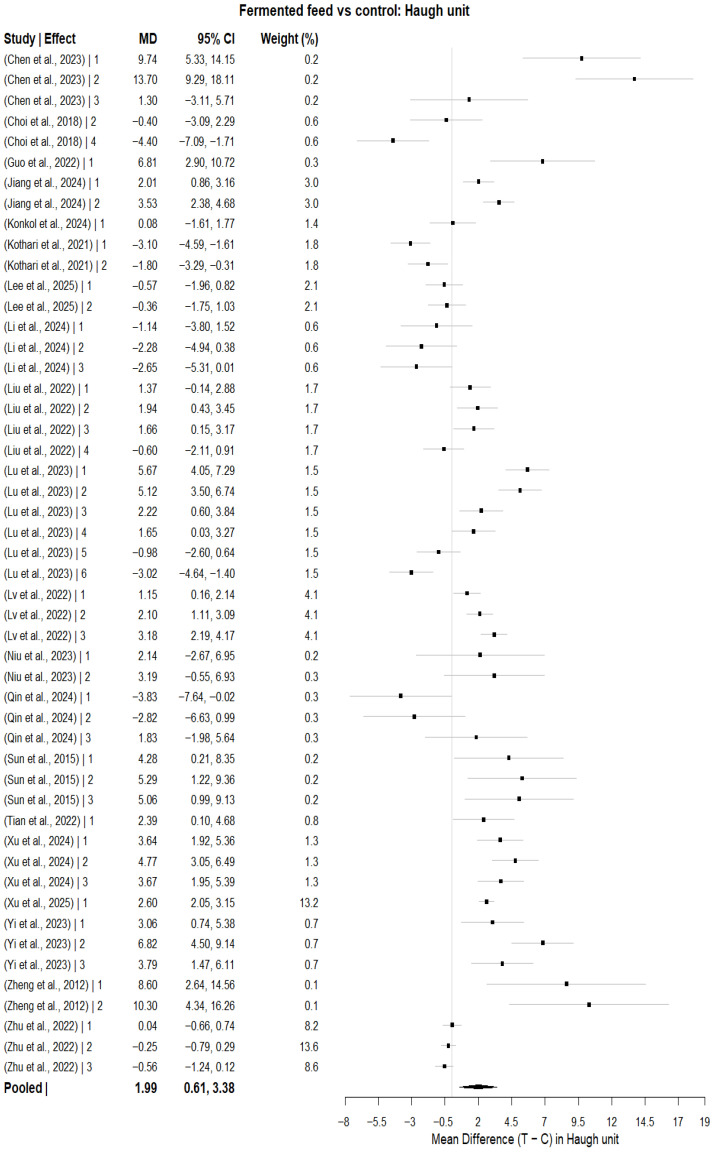
Forest plot showing pooled mean differences (MD) and 95% confidence intervals for the effect of fermented feed supplementation on Haugh unit in laying hens under a multilevel random-effects model [20,46,47,48,49,51,52,53,55,56,57,58,59,60,63,64,65,67]. Squares indicate the effect size estimate for individual studies (with the area of squares proportional to study weight), and horizontal lines indicate corresponding 95% confidence intervals. The diamond represents the pooled effect estimate.

For the Haugh unit, the multilevel random-effects meta-analysis was based on k = 50 effect sizes and produced a statistically significant pooled mean difference (MD = 1.99, SE = 0.71), z = 2.82, *p* < 0.0048; the confidence interval was between 0.61 and 3.38, as presented in Figure 8. The residual heterogeneity was statistically significant (Q = 467.96; df = 49; *p* < 0.0001). The estimated variance components were τ^2^_between-study (StudyID) = 7.53 and τ^2^_within-study (StudyID/EffectID) = 3.28, resulting in a total of 10.82. The corresponding I^2^ estimates were 84.40% (based on the mean sampling variance) and 93.36% (based on the median sampling variance), respectively, indicating substantial heterogeneity. Heterogeneity decomposition revealed that 69.65% of the total heterogeneity was attributable to between-study differences, and 30.35% was attributable to within-study (effect-level) variability. The high I^2^ values indicate substantial between-study variability, suggesting that pooled estimates should be interpreted cautiously. There was no statistically significant meta-regression model including duration, dietary inclusion level, and total number of hens for the effect size.

When fermented feed type was used as a categorical moderator, there was a significant difference among feed types (QM = 51.44, df = 9, *p* < 0.0001); however, high residual heterogeneity remained (QE = 62.56, df = 21, *p* < 0.0001) (k = 31 effect sizes). Pooled mean differences were significantly higher for fermented *Artemisia annua* residues (MD = 4.81, 95% CI: 1.07 to 8.55, *p* = 0.0117), fermented soybean meal (MD = 4.59, 95% CI: 0.98 to 8.21, *p* = 0.0128), red yeast rice (MD = 5.13, 95% CI: 0.92 to 9.34, *p* = 0.0169), Shuanghuanglian fermentation liquor (MD = 4.28, 95% CI: 0.65 to 7.91, *p* = 0.0207) and tea residue-fermented feed (MD = 8.50, 95% CI: 4.18 to 12.82, *p* = 0.0001) compared with the respective reference feed types, whereas other fermented feed types had no statistically significant differences.

**Figure 9:**
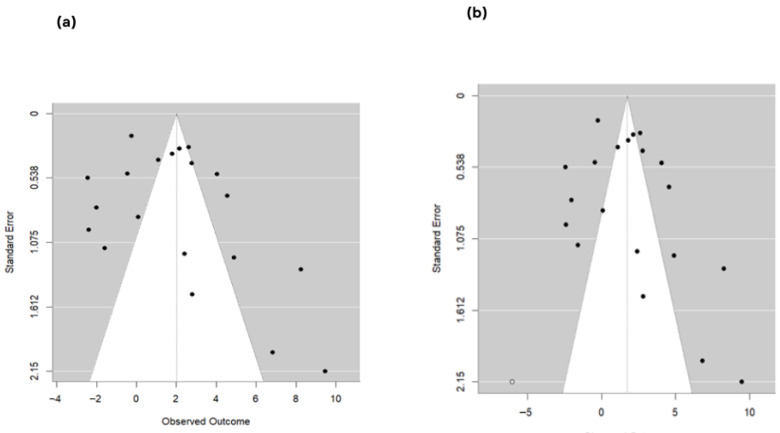
(**a**) Funnel plot of study-level aggregated effect sizes for Haugh unit, illustrating the relationship between observed outcomes and standard error to assess potential publication bias; (**b**) trim-and-fill-adjusted funnel plot showing the imputed study and the corresponding adjusted symmetry. Each dot represents the effect size of an individual study. The pooled effect estimate is represented by the vertical dotted line, and the shaded triangular region represents the expected 95% confidence limits around the pooled estimate.

The pooled estimates were stable, ranging from 1.64 to 2.23, with all 95% CI limits above zero in a leave-one-study-out sensitivity analysis, reflecting stability of the overall effect. Assessment of publication bias at the level of aggregated effects showed funnel plot asymmetry (Figure 9a) according to Egger’s regression test (z = 2.47, *p* = 0.0134); however, trim-and-fill analysis indicated only one potentially missing study (Figure 9b), and the adjusted pooled estimate remained significant (MD = 1.74, 95% CI: 0.29 to 3.18, *p* = 0.019), suggesting that small-study effects did not materially affect the main findings.

#### 3.6.2. Egg Shell Thickness

**Figure 10:**
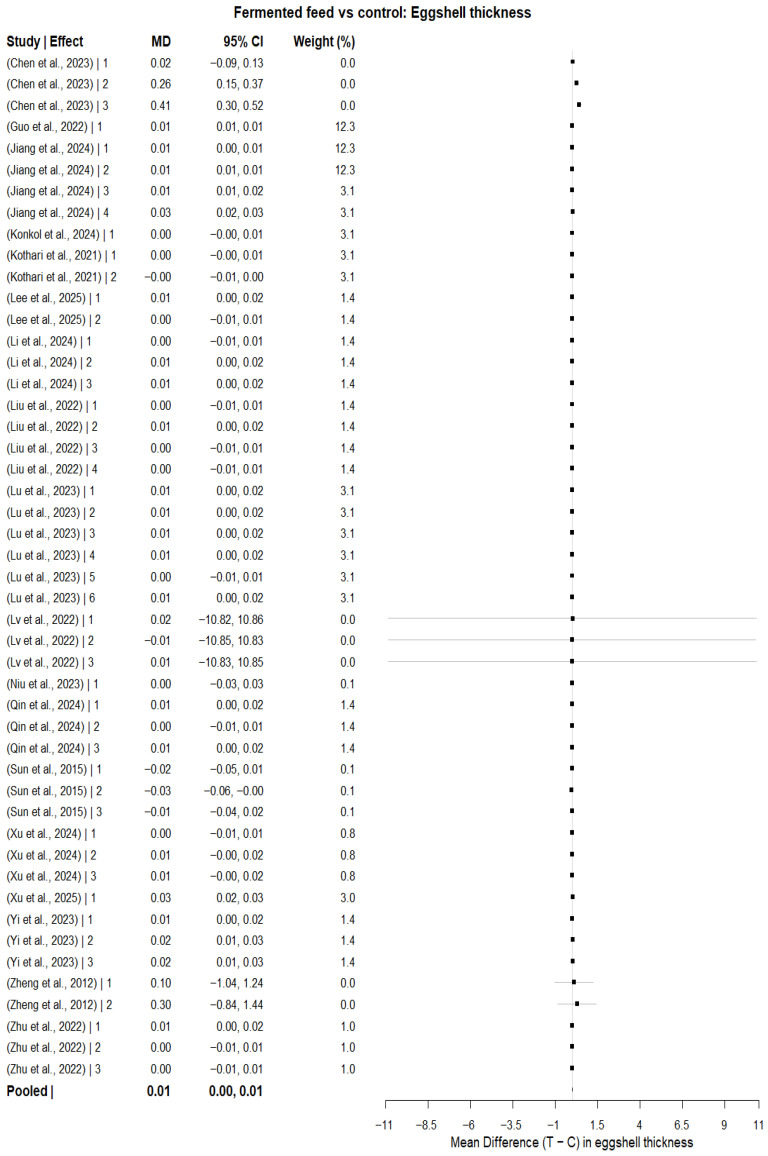
Forest plot showing pooled mean differences (MD) and 95% confidence intervals for the effect of fermented feed supplementation on eggshell thickness in laying hens under a multilevel random-effects model. Squares indicate the effect size estimate for individual studies (with the area of squares proportional to study weight), and horizontal lines indicate corresponding 95% confidence intervals. The diamond represents the pooled effect estimate [20,46,47,48,49,51,52,53,55,56,57,58,59,63,64,65,66,67].

**Figure 11:**
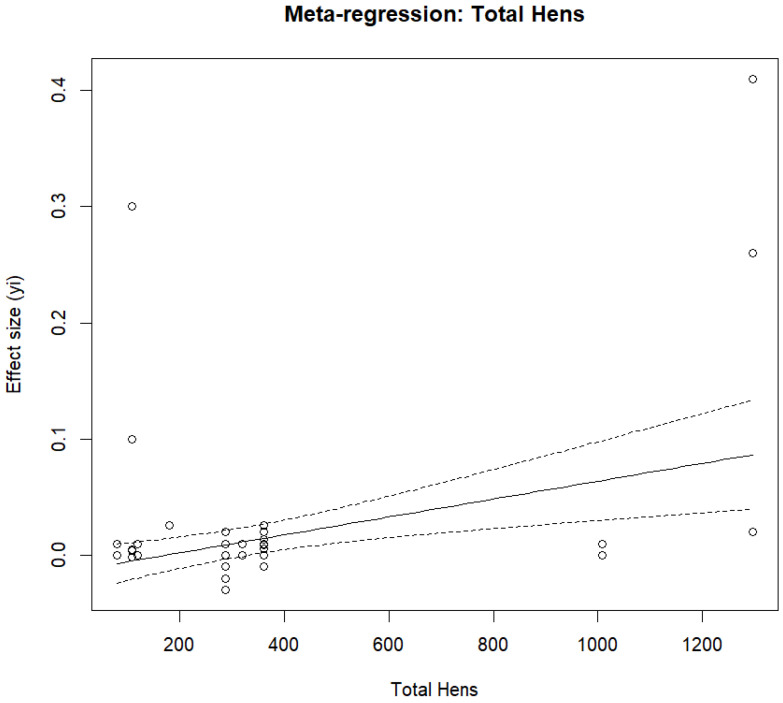
Meta-regression plot depicting the association between total number of hens and effect size (mean difference) for eggshell thickness in laying hens, with the fitted regression line and 95% confidence bands from the multilevel random-effects model. Each circle represents an individual study effect size. The solid line represents the fitted meta-regression line, and the dashed lines indicate the 95% confidence bands around the regression estimate from the multilevel random-effects model.

For eggshell thickness (mm), the multilevel random-effects meta-analysis (REML) including k = 48 effect sizes produced a statistically significant pooled mean difference of 0.0081 mm (SE = 0.0022), with z = 3.65, *p* = 0.0003, and a 95% CI of 0.0037 to 0.0124, as shown in Figure 10. Heterogeneity was statistically significant (Q = 243.97, df = 47, *p* < 0.0001), with small estimated variance components at the between-study level (StudyID: τ^2^ ≈ 5.1 × 10^−5^) and within-study level (StudyID/EffectID: τ^2^ ≈ 2.4 × 10^−5^), yielding a total τ^2^ ≈ 7.5 × 10^−5^; heterogeneity decomposition indicated 68.34% of the modeled heterogeneity was attributable to between-study variability, and 31.66% was attributable to within-study variability. The approximate I^2^ depended on the choice of typical sampling variance (vi), with a value of 0% obtained using mean vi but 80.65% obtained using median vi, indicating substantial inconsistency when using a robust typical vi. The high I^2^ values indicate substantial between-study variability, suggesting that pooled estimates should be interpreted cautiously. Meta-regression showed that total hens was a significant moderator in a linear model (QM = 10.43, df = 1, *p* = 0.0012; positive association), and a quadratic specification was also significant (QM = 18.64, df = 2, *p* < 0.0001) with a significant TotalHens^2^ term (*p* = 0.0038), suggesting nonlinearity in the association between total hens and effect size, as shown in Figure 11.

**Figure 12:**
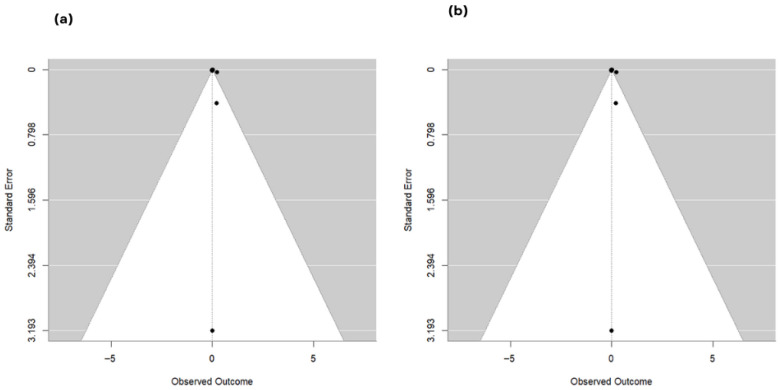
(**a**) Funnel plot of study-level aggregated effect sizes for eggshell thickness, illustrating the distribution of observed outcomes against standard error to assess potential publication bias; (**b**) trim-and-fill–adjusted funnel plot showing no imputed missing studies and preserved symmetry of the effect-size distribution. Each dot represents the effect size of an individual study. The pooled effect estimate is represented by the vertical dotted line, and the shaded triangular region represents the expected 95% confidence limits around the pooled estimate.

Subgroup analysis by fermented feed type (k = 31 effect sizes; REML) showed a statistically significant between-group difference (QM = 24.90, df = 9, *p* = 0.0031), although residual heterogeneity remained significant (QE = 53.55, df = 21, *p* = 0.0001). Relative to the reference feed type, only tea residue-fermented feed showed a statistically significant effect (estimate = 0.2267, SE = 0.0555; 95% CI: 0.1179 to 0.3355; *p* < 0.0001), whereas all other feed-type coefficients were non-significant (*p* > 0.05). Leave-one-study-out sensitivity analysis produced pooled estimates ranging from 0.00696 to 0.01319, with several exclusions yielding confidence intervals that crossed zero, indicating that statistical significance was not fully robust to removal of certain individual studies. Assessment of publication bias using study-level aggregated effects suggested borderline funnel plot asymmetry (Figure 12a) by Egger’s regression test (z = 1.93, *p* = 0.0542); however, trim-and-fill imputed no missing studies (Figure 12b), and the adjusted pooled estimate remained statistically significant (MD = 0.0081; 95% CI: 0.0062 to 0.0100; *p* < 0.0001).

## 4. Discussion

The present findings indicate that fermented feed supplementation represents a promising but context-dependent nutritional strategy for improving laying performance and internal egg quality, with effects varying according to substrate characteristics and implementation conditions. The fermented feed supplementation was associated with a statistically significant increase in egg-laying rate (MD = 2.11 percentage points) and Haugh unit (MD = 1.99), along with a small but significant increase in eggshell thickness (MD = 0.0081 mm), whereas no significant overall effect was observed on feed-to-egg ratio. Although the overall pooled effect was not statistically significant, the high heterogeneity and significant dose-dependent moderator effect indicate that feed efficiency responses to fermented feed are context-dependent and influenced by inclusion level rather than uniformly absent. However, outcome-specific moderator and subgroup analyses revealed that response variability in different types of fermented feed was dependent, and feed efficiency was dependent on dietary inclusion level. Significant heterogeneity was identified, indicating that fermented-feed impacts varied in level and consistency across interventions and experimental contexts. Such heterogeneity is biologically expected in fermented-feed studies, as substrates differ markedly in nutrient composition and anti-nutritional factors; fermentation processes vary in microbial strains, temperature, and duration; and the resulting bioactive metabolites can differ substantially across preparations. Additionally, variation in hen strain, management conditions, and supplementation levels likely contributes to between-study variability. Importantly, “fermented feed” does not represent a biologically uniform intervention but rather a heterogeneous class of substrates and fermentation processes. Substrate origin, microbial consortia, enzymatic activity, fermentation duration, and metabolite profiles vary substantially across studies. These differences imply potentially distinct biological mechanisms of action, including modulation of mineral bioavailability, antioxidant status, gut microbiota composition, nutrient digestibility, and immunometabolic pathways. Therefore, pooled estimates should be interpreted as average effects across mechanistically diverse interventions rather than reflecting a single underlying biological pathway. Although subgroup analyses by feed type were performed, residual heterogeneity suggests that mechanistic diversity remains incompletely captured within aggregated models.

The high egg-laying rate in this meta-analysis indicated that feed fermentation supplementation consistently improved laying productivity, and the consistency of the leave-one-study-out results further strengthened the result. This effect was consistent with other studies from controlled feeding trials, where better laying was observed when fermented feed was added. reported significantly increased laying rates in late-cycle laying hens supplemented with fermented feed, along with reduced broken egg rate and improved feed-to-egg ratio, indicating concurrent improvements in productivity and efficiency. Jia, Lu [68] reported improved laying rate at a 2% inclusion level of fermented distiller grains, which aligns with the dose-dependent pattern observed in our moderator analysis, indicating that supplementation effects vary according to inclusion level rather than increasing uniformly across all doses. Notably, the subgroup analysis showed that the effect was not homogeneous when pooled across the types of fermented feeds. There were statistically significant positive subgroup effects in fermented *Artemisia annua* residues, spent ginger yeast cultures, and Shuanghuanglian fermentation liquor, but not in other types of fermented feeds. The pronounced response observed with fermented *Artemisia annua* residues may be attributed to its rich phytochemical profile, including flavonoids and other bioactive compounds with antioxidant and antimicrobial properties. Fermentation may enhance the bioavailability of these compounds while reducing anti-nutritional factors, potentially improving gut health, nutrient utilization, and overall laying performance. However, the magnitude of response is also likely influenced by the microbial composition and fermentation conditions employed. Different starter cultures (e.g., yeast, lactic acid bacteria, or mixed consortia), fermentation duration, and temperature can substantially alter enzymatic activity, metabolite production, and nutrient transformation. Such process-related factors may modify the concentration of bioactive compounds and the digestibility of substrates, thereby contributing to variability in observed performance outcomes beyond feed type alone. This trend was quite similar to study level results with fermented *Artemisia annua* residues, Yi, He [65] in which obtained significantly higher laying rates at 2% and 4% inclusion levels. Similarly, SGYCs significantly enhanced laying rate at an optimal dose during the experiment conducted by Liu, Jin [53], supporting the specificity of feed type identified in the meta-analysis. These differential responses may reflect substrate-specific bioactive compounds and fermentation-derived metabolites that enhance nutrient digestibility, gut microbial balance, and antioxidant capacity. For example, *Artemisia annua* and herbal fermentation liquors contain phytochemicals and secondary metabolites that may modulate intestinal function and systemic metabolic efficiency, thereby supporting improved laying performance. Although inclusion level, trial duration, and total hens were not identified as statistically significant moderators of laying rate, these findings should be interpreted cautiously considering the substantial heterogeneity observed across studies. The absence of statistically significant moderator effects does not necessarily indicate a lack of influence but rather suggests that their impact was not consistently detectable within the available dataset and may have been obscured by between-study variability. While significant subgroup differences were observed across fermented feed types, this pattern should also be interpreted with caution, as residual heterogeneity indicates that multiple intervention-related and contextual factors likely contribute to response variability. Overall, fermented feed supplementation was associated with improved laying rate; however, the magnitude of improvement appears to vary according to specific intervention characteristics and study conditions.

Unlike the egg-laying rate, the pooled estimate of the feed-to-egg ratio was not statistically significant, meaning that there was no uniform increase in feed efficiency of trials when using fermented feed supplements. Nevertheless, the non-significant pooled outcome was best explained by the significant level of heterogeneity and the clear indication that the effect sizes varied systematically by inclusion level and type of fermented feed. Several individual studies also showed an increase in feed efficiency, consistent with the direction of effects within key subgroups. Guo, Xu [46] reported a substantial drop in the feed-to-conversion ratio, and Yi, He [65] also reported a much lower feed-to-egg ratio at 2% fermented *Artemisia annua* residues. Liu, Jin [53] also found a reduction in feed ratio in favor of the SGYC supplement at the most effective dose, indicating better feed utilization under certain intervention conditions. These findings suggest that improvements in feed efficiency may be linked to enhanced nutrient digestibility and improved gastrointestinal function resulting from fermentation-induced degradation of anti-nutritional factors and increased availability of bioactive metabolites. Fermentation processes may promote enzymatic breakdown of complex feed components, improve amino acid and mineral bioavailability, and modulate gut microbiota composition, collectively contributing to more efficient feed utilization under optimal inclusion levels. The moderator analysis offered a mechanistic explanation for why a positive trial-level result did not translate into a generally significant pooled effect. The meta-regression found that dietary inclusion level was a statistically significant moderator, indicating that responses to the feed-to-egg ratio were dose-dependent. Also, subgroup analysis showed that fermented *Artemisia annua* residues, SGYCs, and Shuanghuanglian fermentation liquor significantly reduced the feed-to-egg ratio; other types of fermented feed did not. This trend suggests that better feed efficiency depends on selecting specific fermented feeds and achieving adequate inclusion levels, rather than on the overall characteristics of fermented feeds. This interpretation was further supported by the multivariable model, in which inclusion level remained a significant predictor, though the effects of feed type persisted even after dosage was considered. Hence, the overall non-significant pooled effect was best consistent with a situation in which there were significant feed efficiency gains in some prescribed fermented-feed subtypes and dose ranges, offset in the aggregate by non-homogeneous interventions that yielded weaker or no responses.

The statistically significant positive change in the Haugh unit in this meta-analysis indicated a general improvement in albumen quality resulting from fermented feed supplementation. This observation was highly consistent with several feeding trials that had recorded improved albumen-related indices with the inclusion of fermented feed. The pooled direction and magnitude of effect were supported by higher albumen height and Haugh unit values in late-cycle hens fed fermented feed [46]. Obianwuna, Huang [21] found that fermented soybean meal supplementation had a notable effect on albumen height and Haugh unit improvement, indicating that fermented protein-based ingredients can improve internal egg quality. This trend was also supported by evidence of other fermented interventions. Sun, Wu [59] found a greater increase in Haugh units as graded supplementation of red yeast rice was made, and Chen, Zhou [20] found that a significant improvement in Haugh units occurred with moderate levels of inclusion of tea residue-fermented feed. The improvement in albumen quality may be biologically attributable to enhanced protein digestibility and amino acid availability following fermentation, as well as improved intestinal absorption efficiency. Fermentation reduces anti-nutritional factors and may increase the availability of small peptides, enzymes, and bioactive compounds that support oviduct function and albumen protein synthesis. Additionally, modulation of gut microbiota and antioxidant status may contribute to improved internal egg quality and albumen structural integrity. The feed-type subgroup outcomes provided greater clarity, showing that the gain in Haugh unit was particularly pronounced in fermented *Artemisia annua* residues, fermented soybean meal, red yeast rice, Shuanghuanglian fermentation liquor, and fermented feed using tea residues. This was in line with Yi, He [65], who found a significant increase in Haugh unit at 2% fermented *Artemisia annua* residues, and Chen, Zhou [20], who achieved improved Haugh unit at 1% and 3% tea residue fermented feed. Interestingly, inclusion level, trial duration, and total hens were not found to be important moderators of this effect in the meta-regression, suggesting that the observed heterogeneity was more closely linked to intervention attributes than to simple trial features. Even though Egger’s regression suggested funnel plot Asymmetry, trim-and-fill imputed only one study that was missing, and the adjusted pooled estimate remained statistically significant. Fermented feed supplementation improved the quality of albumen, and this correlation remained strong even after accounting for potential small-study effects.

The meta-analysis reported a small but significant difference in eggshell thickness in response to fermented feed supplementation, suggesting improved shell quality. This result was consistent with evidence from trials showing that fermented feeds can improve eggshell quality under certain conditions. Chen, Zhou [20] also found the eggshell to become significantly thicker for 3% and 5% tea residue-fermented feed, which aligns directly with our subgroup finding that tea residue-fermented feed was the only product type associated with a statistically significant effect on eggshell thickness compared to the reference. Shin, Park [69] also reported that the addition of germinated and fermented unmarketable soybeans significantly increased eggshell thickness, indicating the potential for fermented soybean-based treatments to improve shell quality. The observed improvement in shell thickness may be biologically linked to enhanced mineral bioavailability, particularly calcium and phosphorus, following fermentation-induced degradation of phytates and other anti-nutritional factors. Fermentation may also improve intestinal absorption efficiency and alter gut microbial activity, thereby facilitating better mineral utilization for shell formation in the uterus. Additionally, certain fermented substrates may contain bioactive compounds that support calcium metabolism and shell matrix protein deposition. However, the robustness of our findings on eggshell thickness was lower than that of other results; confidence intervals from several leave-one-out exclusions included zero. This diminished stability was consistent with the heterogeneous evidence at the study level, as several studies reported that eggshell thickness did not differ markedly. For example, Yi, He [65] found no significant differences in eggshell thickness between hens fed fermented *Artemisia annua* residues, and such null effects may have contributed to the heterogeneity and reduced statistical significance observed for this outcome. The meta-regression result that the total number of hens significantly moderate eggshell thickness also indicated that the study scale might have contributed to the estimated effects, possibly owing to differences in management conditions, rigor, or measurement precision across trials. Although Egger’s test was borderline and trim-and-fill did not impute any missing studies, the lower stability in the sensitivity analyses suggested that we should treat eggshell thickness more hesitantly than the egg-laying rate and Haugh units.

The high heterogeneity in outcomes indicated that the effects of fermented feed were not homogeneous and depended significantly on the nature of the interventions. Fermented feed type accounted for a significant amount of between-effects variance in the attributes of egg-laying rate and Haugh unit, and individual interventions (fermented *Artemisia annua* residues, SGYCs, Shuanghuanglian fermentation liquor, fermented soybean meal, red yeast rice, and tea residue-fermented feed) demonstrated more uniform advantages. In the case of the feed-to-egg ratio, the level of inclusiveness was also a statistically significant moderator, suggesting the dose dependence was a salient characteristic of the responses to feed efficiency. Regarding eggshell thickness, the strong correlation with total hens indicated that the study’s scale and its correlated design and management characteristics might have contributed to the results. These patterns of moderation led to the conclusion that the application of fermented feed supplementation could not be considered a homogeneous intervention; instead, the outcome seemed to depend on the fermented substrate, fermentation properties, and the inclusion level used.

The strength of this meta-analysis included outcome-specific multilevel modeling to control dependence among multiple effect sizes within studies, and the application of study-level methods to assess publication bias and minimize unit-of-analysis error. Nonetheless, several limitations were evident. A limitation of this systematic literature review and meta-analysis is that full-text screening was restricted to studies with accessible full-text availability, which may introduce selection bias and limit comprehensiveness. Further unmeasured trial-level effects likely influenced the results, as considerable heterogeneity persisted even after stratification by fermented feed type. Second, the fermented feed interventions used a variety of substrates and fermentation techniques, limiting direct comparisons and likely leading to residual heterogeneity. This diversity reflects not only methodological variability but also fundamental biological differences between fermented substrates, which may operate through distinct mechanistic pathways. Third, evidence of publication bias was identified for the Haugh unit, as indicated by funnel plot asymmetry and a statistically significant Egger’s regression test, although trim-and-fill adjustment reduced but did not eliminate the observed effect. The reduction in pooled magnitude after adjustment suggests that small-study effects may have partially inflated effect estimates, particularly for albumen quality outcomes. For eggshell thickness, borderline funnel asymmetry and sensitivity of the pooled estimate to exclusion of individual studies further indicate that some findings may be influenced by study-level effects or limited-study precision. Finally, robustness was outcome-specific, as eggshell thickness showed lower stability across the leave-one-out analyses. Taken together, the presence of substantial heterogeneity, feed-type specificity, dose-dependent responses, and potential small-study influence indicates that effect magnitudes should be interpreted cautiously, even when statistical significance is retained.

Beyond quantifying pooled effects, this meta-analysis provides a structured comparison of diverse fermented feed strategies across controlled trials, enabling identification of feed-type specificity, dose-dependent responses, and key sources of heterogeneity that are not evident in individual studies. By synthesizing fragmented evidence, this work clarifies which fermented substrates demonstrate more consistent associations with performance and egg quality outcomes and highlights the importance of standardized reporting of fermentation protocols, substrate composition, baseline productivity, and inclusion levels. These findings offer practical guidance for future experimental design, including optimization of dosage ranges, clearer characterization of fermentation processes, and improved methodological rigor. Thus, this synthesis contributes not only quantitative estimates but also a strategic framework for advancing research on fermented feed supplementation in laying hens.

## 5. Conclusions

This meta-analysis demonstrated that fermented feed supplementation offered promising yet complex advantages for laying hen performance and egg quality, with effectiveness significantly influenced by the type of fermented feed and application strategy, rather than being a uniform nutritional intervention. Fermented feed supplementation contributed to a statistically significant enhancement in egg-laying rate and a consistent improvement in albumen quality, indicated by elevated Haugh unit values. Additionally, a modest yet significant increase in eggshell thickness was noted, though with reduced robustness in sensitivity analyses. The overall effect on the feed-to-egg ratio was not statistically significant, indicating considerable variability across studies, primarily driven by feed-type-specific responses and dose-dependent effects, as elucidated by moderator and subgroup analyses. Fermented residues of *Artemisia annua*, spent ginger yeast cultures, fermented soybean-based feeds, Shuanghuanglian fermentation liquor, tea residue-fermented feed, and red yeast rice consistently produced superior results compared to other fermented feed types, highlighting the necessity of selective intervention over generalized use of fermented feeds. This meta-analysis faced inherent challenges, including variability in methodological quality across primary studies, diversity in fermentation substrates and protocols, and the potential impact of publication bias, which may have compromised the accuracy of pooled estimates, especially for egg quality outcomes. This study enhanced the current literature by integrating multilevel modeling with thorough subgroup and moderator analyses, demonstrating that the advantages of fermented feed for performance and egg quality were specific to feed type and environment, rather than being binary. The findings offered practical recommendations for layer nutrition, indicating that prioritizing well-supported fermented feed varieties and maximizing inclusion levels could enhance productivity and quality outcomes. Future standardized, high-quality trials are necessary to elucidate dose–response relationships and expand the database for tailored fermented feed solutions in commercial laying hen production.

## Data Availability

All relevant data are within the manuscript.

## Abbreviations

Abbreviation	Full Form
*A. niger*	*Aspergillus niger*
*A. oryzae*	*Aspergillus oryzae*
AAR	*Artemisia annua* Residues
ADFI	Average Daily Feed Intake
*B. subtilis*	*Bacillus subtilis*
CBT^®^	*Chlorella vulgaris* CBT
*C. butyricum*	*Clostridium butyricum*
CHM	Chinese Herbal Medicine
CI	Confidence Interval
CON	Control
C_Mean	Control Mean
C_n	Control Sample Size
C_SE	Control Standard Error
df	Degrees of Freedom
FCR	Feed Conversion Ratio
FCSM	Fermented Cottonseed Meal
FPNE	Fermented Pine Needle Extract
FRSM	Fermented Rapeseed Meal
I^2^	I-squared (heterogeneity statistic)
Incl	Inclusion Level
Incl^2^	Quadratic Term of Inclusion Level
Incl_c	Centered Inclusion Level
k	Number of Effect Sizes
*L. crispatus*	*Lactobacillus crispatus*
*L. plantarum*	*Lactobacillus plantarum*
*L. salivarius*	*Lactobacillus salivarius*
LRT	Likelihood Ratio Test
MD	Mean Difference
MeSH	Medical Subject Headings
n	Sample Size
NR	Not Reported
PRISMA	Preferred Reporting Items for Systematic Reviews and Meta-Analyses
Q	Cochran’s Q statistic
QE	Residual Q Statistic (Test of Residual Heterogeneity)
QM	Model Q Statistic (Test of Moderators)
R	R Software
RCT	Randomized Controlled Trial
REML	Restricted Maximum Likelihood
RoB	Risk of Bias
*S. cerevisiae*	*Saccharomyces cerevisiae*
SBM	Soybean Meal
SD	Standard Deviation
SE	Standard Error
SEM	Standard Error of the Mean
SFL	Shuanghuanglian Fermentation Liquor
SGYCs	Spent Ginger Yeast Cultures
SYRCLE	Systematic Review Centre for Laboratory Animal Experimentation
TITLE-ABS-KEY	Title-Abstract-Keyword
TotalHens^2^	Quadratic Term of Total Number of Hens
TS	Topic Search
T_Mean	Treatment Mean
T_n	Treatment Sample Size
T_SE	Treatment Standard Error
tiab	Title/Abstract
τ^2^	Tau-squared (Between-study Variance)
τ^2^_between-study	Between-Study Variance Component
τ^2^_within-study	Within-Study Variance Component
URSM	Unfermented Rapeseed Meal
vi	Sampling Variance
WOS	Web of Science
z	Z Statistic
